# Editorial: Circadian biology, cardiovascular function and disease

**DOI:** 10.3389/fphar.2023.1132735

**Published:** 2023-01-13

**Authors:** Nelson Chong, Paola Campagnolo

**Affiliations:** ^1^ School of Science and Technology, Nottingham Trent University, Nottingham, United Kingdom; ^2^ School of Biosciences and Medicine, University of Surrey, Guildford, United Kingdom

**Keywords:** circadian rhythms, cardiovascular diseases, cardiac arrhythmias, myocardial infarction, chronobiology, cardioprotection and ischemia-reperfusion injury, chronotherapy & chronotherapeutics, clock gene expression

Cardiovascular disease (CVD) are complex multi-factorial disorders that constitutes the leading cause of morbidity and mortality worldwide (Roth et al., 2020). Homeostatic heart function depends on several key physiological parameters such as cardiac contractility, metabolic state and overall effective hemodynamic regulation of the body (Verbrugge et al., 2020).

Mammalian circadian (∼24 h) clocks are ubiquitous autoregulatory molecular interactive transcriptional/translational feedback loops that precisely drive diverse daily physiological processes. Centrally, the suprachiasmatic nucleus (SCN) in the brain respond to light entrainment, and act as a pacemaker of the circadian timing in the body. At a molecular level, each cell possesses clock genes which are regulated by a variety of SCN-derived periodical stimulants and act as peripheral clocks. Heterodimers of central clock factors (CLOCK and BMAL1) bind to E-boxes within diverse target gene promoters including the clock genes Per and Cry. PER and CRY then dimerize and repress their own transcription by inhibiting CLOCK:BMAL1. CLOCK:BMAL1 also induce the expression of REV-ERBα, which represses Bmal1 transcription (Takahashi, 2017). The precise orchestration of this interlocking feedback loop regulates many fundamental aspects of cardiovascular processes such as daily rhythms in blood pressure and heart rate, electrophysiology, cardiac metabolism and contractility, endothelial cell biology, platelet aggregation, and thrombus formation are also influenced by the local intrinsic circadian clock. Circadian misalignment or clock dysfunction can lead to diverse cardiovascular diseases such as cardiac arrhythmias, cardiomyopathy and diabetes (Martino and Young, 2015; Alibhai et al., 2017; Thosar et al., 2018; Chellappa et al., 2019; Crnko et al., 2019; Rana et al., 2020). The occurrence of adverse cardiovascular events such as acute heart failure, myocardial infarction, stroke, and cardiac arrhythmias, also exhibits a daily variation with prevalence in the early morning hours (Cohen et al., 1997).

The purpose of this Research Topic is to highlight the impact of the circadian axis on the regulation of cardiac function and disease, including its interaction with feeding behaviour, electrophysiology, pathogenesis of cardiac disease and mechanisms of cardio-protection and repair.

Schroder and Delisle reviewed the impact of feeding behaviour on circadian cardiac physiology. Restricted feeding studies have been used to disentangle the interconnections between the cellular circadian clocks, physiological circadian rhythms (such as change in heart rate), and food (nutrient) intake (Rana et al., 2020; Chellapa et al., 2021). By restricting feeding time in mice, it has emerged the SCN and the molecular clock (driven by *Bmal-1*) are dispensable for the entrainment of cardiac function, and a new player -food entertainable oscillator (FEO)- may instead be a crucial determinant of circadian cardiac physiology, such as heart rate. Deciphering the impact of feeding behaviour on human cardiovascular homeostasis may provide a novel avenue for novel approaches to combat CVD risk.

The importance of circadian rhythms on the cardiac electrophysiology has also been reviewed by Seed and Hearn who reported on the existing literature on clinical trials investigating Long QT syndrome, revealing a chronic lack of time-of-day information. Given the known the diurnal variation in QT interval, this may affect the electrocardiogram measurements and the trial outcomes. The authors also provide a tool to help incorporating the chronobiology aspect into the design of clinical trials.

Two papers reported intriguing findings on the role circadian genes in the post-ischemic repair. In the study by Bao et al. Verapamil, an activator of SIRT1 (Silent information regulator of transcription 1), positively influenced the recovery after ischemia/reperfusion. SIRT1 is a key regulator of the circadian clock and is required for high-magnitude circadian transcription of several core clock genes and is expressed in a circadian manner. This paper demonstrates that Verapamil reduces damage in cultured cardiomyocytes exposed to hypoxia/reperfusion and animal models of ischemia/reperfusion, through its activation of SIRT-1 dependent antioxidant mechanism. It is exciting to speculate that its circadian regulatory effect may also be involved. Indeed, the study by Kilgallen et al. investigated the role of the circadian rhythm in the acute immune response occurring immediately after myocardial infarction. By inducing cardiac ischemia at different Zeitgeber Times (ZT) and studying the effects at a very early time point (3 h), they found that animals undergoing surgery at the early active phase (ZT14-ZT20) displayed an increase of circulating chemokines involved in neutrophil chemotaxis and activation in mice undergoing surgery, and a concomitant reduction of circulating neutrophils, suggesting homing to the heart. This was accompanied by an increase in cardiac Troponin I, a circulating marker of cardiac damage. Indeed, increase in neutrophil infiltration at the site of ischemia has previously been linked with worse outcome (Schloss et al., 2016). Kilgallen et al. paper provides a new insight in the mechanisms underlying the unfavourable consequences of early morning cardiac events in patients, which are known to result in large damage and lower recovery rates (Martino and Young, 2015).

Post-ischemic cardiac repair is also dependent on efficient angiogenesis, the formation of new blood vessels, to restore blood flow to the affected areas. Mastrullo et al. investigated the importance of the circadian clock in pericytes, perivascular cells that play a critical role in angiogenesis and regeneration (Campagnolo et al., 2010) and constitute a promising source of cells for cell therapy and tissue engineering. Pericyte presented a rhythmic expression of circadian genes which are crucial to their viability and were capable of synchronising endothelial cells in co-culture. Importantly, formation of complete blood vessels (presenting organised structures of endothelial cells surrounded by pericytes) in 3D tissue-engineered scaffolds was affected by circadian genes ablation and was increased by cell synchronisation. This indicates that the circadian clock cannot be ignored when planning revascularizing interventions in the heart.

When myocardial ischemia is instead unresolved, compensatory mechanisms of cardiomyocyte hypertrophy and fibrosis ensue to preserve mechanical stability and cardiac output, often resulting in a chronic insufficiency termed cardiomyopathy (Nakamura and Sadoshima, 2018). Sonkar et al. investigated the role of the circadian clock in cardiomyopathy, demonstrating that the cardiomyocyte-specific disruption of *Bmal-1* gene determined increased the pathological remodelling. The mechanism is driven by an increased growth hormone (GH) sensitivity, leading to the induction of insulin-like growth factor 1, and knockdown of the GH receptor normalised the aberrant phenotype.

Heart fibrosis observed in cardiomyopathy, but also in pressure-overload models of cardiac failure, is driven by the differentiation and proliferation of (myo)fibroblasts which are responsible for the increased extracellular matrix deposition (Frangogiannis, 2021). Luo et al. investigated two REV-ERB knockout lines of fibroblasts *in vitro* and found that the knockout had no effect of mouse embryonic fibroblasts but it significantly increased the differentiation of cardiac fibroblasts. Furthermore, treatment of activated cardiac fibroblast with SR9009 (REV-ERB agonist) reduced the differentiation into myofibroblasts, but this mechanism was REV-ERB independent. It is interestingly to observe, therefore, how the clock genes manipulation in two adjacent cardiac cell types (cardiomyocytes and fibroblasts) both drive an increase in fibrosis, suggesting that use of pharmacological intervention could deliver a synergistic effect.
